# Study on the enhancement of volatile components extraction from *Lysimachia foenum-graecum* Hance essential oil by metal salts

**DOI:** 10.3389/fchem.2026.1869613

**Published:** 2026-07-07

**Authors:** Qingfu Wang, Jiaming Zhao, Zening Wang, Liuke Zhang, Lei Li, Yibo Ning, Zhipeng Jing, Pengfei Yang, Yongzhen Zhao, Changtong Lu

**Affiliations:** 1 Technology Center, China Tobacco Henan Industrial Co., Ltd., Zhengzhou, Henan, China; 2 College of Tobacco Science and Engineering, Zhengzhou University of Light Industry, Zhengzhou, China

**Keywords:** comprehensive two-dimensional gas chromatography-mass spectrometry, *Lysimachia foenum-graecum* Hance essential oil, metal salt intensification, multivariate statistical analysis, volatiles

## Abstract

To investigate the effects of various metal salt solutions on the extraction efficiency of *Lysimachia foenum-graecum* Hance essential oil (LHEO), steam distillation coupled with comprehensive two-dimensional gas chromatography mass spectrometry was applied for volatile characterization. Single-factor experiments and orthogonal experimental design were adopted to evaluate four metal salts (FeCl_3_, MgCl_2_, MnCl_2_, ZnCl_2_). All four metal salts significantly improved essential oil yield with a maximum increase of 106%, and the optimal intensification condition was determined as 1.2% ZnCl_2_ with 24 h extraction. A total of 235 compounds were detected in five samples. Orthogonal partial least squares-discriminant analysis revealed obvious sample differences based on 72 common volatiles, and 17 differential compounds including furfural, neophytadiene, phytol, isophytol, α-terpineol, linalool and geraniol were identified according to VIP scores. The findings clarify the influences of different metal salt solutions on LHEO volatile profiles and provide theoretical basis and practical reference for extraction process optimization and quality control.

## 1 Introduction

*Lysimachia foenum-graecum* Hance (LH), is a perennial herb of the Primulaceae family, primarily distributed in the Guangxi, Yunnan, Guangdong, and Guizhou provinces of China (Dai et al., 2023). Previous studies have demonstrated its medicinal properties, including antioxidant, antibacterial, fungicidal, anti-inflammatory, anticancer, etc. (Li et al., 2009; Qin et al., 2024; Widsanusan et al., 2019; Yi et al., 2011). The chemical constituents of LH mainly include volatile components, triterpenes, saponins, flavonoids, polysaccharides. Among these, volatile components serve as the primary material basis for the unique aroma of LH (Shen et al., 2005). As a precious natural spice, LHEO are characterized by their stable and long-lasting aroma, making them highly valued in the tobacco, alcohol, cosmetics, and food industries (Qin et al., 2024).

Regarding the regulation of the release behavior of volatile components in natural fragrances, the introduction of inorganic metal salts into aqueous systems has emerged as a research hotspot for enhancing aroma quality (Baillie et al., 2006; Han et al., 2023). The addition of metal salts not only exerts a significant salting-out effect, which reduces the degree of organic solvation through ion hydration, thereby increasing the thermodynamic activity of volatile components and forcing their transfer to the gas phase, but also alters the surface tension and microenvironment of the system through cations of varying charge densities, leading to differentiated ionic strength effects (Grundl et al., 2017; Ju et al., 2017; Ni et al., 2026; Sriprablom et al., 2019). Steam distillation (SD), with its remarkable advantages of simple operation, low equipment cost and no organic solvent residue in the product, has long been an effective method for the extraction of essential oil from plants (Crescente et al., 2025). Although accepted as the standard Pharmacopoeia protocol, conventional SD relies purely on physical steam driving forces, often suffering from prolonged extraction hours due to its inability to disrupt dense cell walls. Conversely, while ultrasonic extraction improves efficiency via acoustic cavitation, it non-selectively co-extracts massive non-volatile matrices that heavily interfere with subsequent volatile analysis (Zhou et al., 2025). To bridge these gaps, the present study introduces a chemical intensification strategy directly into traditional SD. By incorporating multi-valent metal cations (Zn^2+^, Fe^3+^, Mn^2+^, and Mg^2+^) to leverage selective coordination and salting-out effects, this approach drastically accelerates volatile mass transfer while fully retaining the high product purity inherent to steam distillation. This study uses an orthogonal experimental design to quickly identify key factors (salt type, dosage, extraction time) and their optimal levels with minimal trials, ensuring efficient and reliable results (Qi et al., 2026).

Appropriate detection methods can markedly improve the efficiency and reliability of volatile component analysis (Yang and Cadwallader, 2025). Qin et al. used HS-SPME combined with GC-IMS to identify 29 volatile components in LH from different storage times and origins. Among them, 1,8-cineole, 2-octanone, heptanal monomer, and 2-methylpropa are key for origin identification, while phenylacetaldehyde, 2-ethylfuran, 2-pentyl furan monomer, gamma-butyrolactone monomer, and maltol are key for LH’s stronger fragrance after storage (Qin et al., 2024). Due to the complexity of the volatile constituents in LHEO, the classic one-dimensional gas chromatography (1D-GC) is often interfered with serious peak overlapping. The two-dimensional chromatography-mass spectrometry method (GC × GC-MS) allows for a single run with a sample displaying two independent separations gives a large increase in resolution, peak capacity and sensitivity (Dalluge et al., 2003). With its robust qualitative and quantitative capabilities, this technology has been widely applied in the analysis of complex systems, such as flavors, fragrances, and food products (Delač Salopek et al., 2026; Wang et al., 2026). The paper aims to reveal how metal salts can promote aroma release from natural LHEO through orthogonal process optimization, and systematically assess the effect of salt ions on aroma release. The outcome could provide a scientific basis for the valuable application of natural perfumes.

## 2 Materials and methods

### 2.1 Materials

#### 2.1.1 Sample preparation

The dried LH (Guangxi, China) was provided by Yunnan Plant Pharmaceutical Biotechnology Co., Ltd. The plant material was pulverized into fine powder and passed through a 120-mesh sieve to ensure particle size uniformity. The samples were kept for future use.

#### 2.1.2 Chemical and reagents

Linalool (98%), phenethyl alcohol (99%), myrcenol (98%), nerol (98%), β-eudesmol (98%), (Z)-9-tetradecenol (97%), isophytol (98%), phytol (98%), geranylgeraniol (99%), 3,4,5-trimethoxytoluene (98%), phenol (99.5%), 2-cresol (99%), paracresol (99%), 2-ethylphenol (98%), 2,4-di-tert-butylphenol (98%), diaethylacetal (99.5%), (E,E)-2,4-heptadienal (98%) and decanal (97%) were obtained from yuanye Bio-Technology Co., Ltd., Shanghai, China. Hendecenal (78%), undecanal (98%), dodecanal (98%), tetradecanal (98%), 4-biphenylaldehyde (98%), 1-pentadecanal (98%), isovaleric acid (99%), methylethylacetic acid (99%), octanoic acid (99.5%), hendecanoic acid (99%), pentadecylic acid (97%), palmitic acid (98%), 1-pentadecene (99%), δ-cadinene (95%), α-hexadecene (95%), hexahydroaplotaxene (95%), α-octadecene (95%), neophytadiene (95%), indanone (98%), β-ionone (99%), 2-tetradecanone (97%) and 2-pentandecanone (95%) were gotten from Aladdin Reagents Co., Ltd., Shanghai, China. Fluorenone (99.5%), perhydrofarnesyl acetone (98%), farnesan (98%), phytan (98%), fural (99%), 2-acetylfurane (99%), dibenzofurane (98%), methyl laurate (98%), ethyl laurate (99%), methyl hexadecanoate (99%), methyl linoleate (99%), methyl linolenate (99%), ethyl linolenate (80%), α-terpineol (98%), geraniol (98%), guaiacol (98%), tridecylic acid (98%), telfairic acid (98%), oleic acid (99%), damascenone (98%), 2-tridecanone (98%), copaene (95%), calamenene (95%), linalool oxide (95%) and methyl isocostate (95%) was purchased from TCI Development Co., Ltd., Shanghai, China. C7-C30 alkanes and dichloromethane (HPLC grade) were gotten from J&K Chemicals Ltd., Beijing, China. Sodium sulfate anhydrous was obtained from Sinopharm Chemical Reagent Co., Ltd.

### 2.2 Methods

#### 2.2.1 Extraction of volatile compounds in LHEO

The LHEO was extracted by SD. Briefly, 10.0 g of the prepared powder was taken into a distillation flask and adding 200 mL ultra-pure water (the ratio of water to LH powder is 20:1). Dichloromethane was utilized to take away the distillate. The organic phase was subsequently dehydrated with Na_2_SO_4_ overnight. The crude LHEO was obtained by evaporation under reduced pressure of the solvent after filtration using a rotary evaporator. The yield of the extraction was calculated and all experiments were conducted in triplicate.

#### 2.2.2 Single-factor experiments

The yield of LHEO was investigated using single-factor experiments to assess the impact of salt dosage, metal salt type, and extraction time (Table 1). The experimental variables set in this study were as follows – four types of metal ions that is (Fe^3+^, Mg^2+^, Zn^2+^ and Mn^2+^) were compared, the salt dosage was varied in five levels to be (0.4%, 0.8%, 1.2%, 1.6% and 2.0% w/w), extraction time was investigated in five intervals (12, 16, 20, 24 and 28 h). These experiments were conducted to determine the optimal ranges and central levels for the subsequent orthogonal design.

**TABLE 1:** Single factor levels.

Level	Factors		
	A (salt categories)	B (amount of addition/%)	C(Time/h)
1	MnCl_2_	0.4	12
2	ZnCl_2_	0.8	16
3	FeCl_3_	1.2	20
4	MgCl_2_	1.6	24

#### 2.2.3 Orthogonal experimental design

Based on the preliminary results of the single-factor trials, an L_16_ (4^5^) orthogonal array was employed to optimize the extraction process involving multiple variables. The factors and their corresponding levels were summarized in Table 2. Analysis of Variance (ANOVA) were utilized to evaluate the significance of each parameter and to identify the optimal combination of extraction conditions for maximizing the essential oil yield.

**TABLE 2:** Results of orthogonal experiment and range analysis.

No	Factors					Yield/%
	A	B	C	D (blank column)	E (blank column)	
1	1	1	1	1	1	0.275
2	1	2	2	2	2	0.503
3	1	3	3	3	3	0.666
4	1	4	4	4	4	0.685
5	2	1	2	3	4	0.402
6	2	2	1	4	3	0.456
7	2	3	4	1	2	1.081
8	2	4	3	2	1	0.852
9	3	1	3	4	2	0.662
10	3	2	4	3	1	0.836
11	3	3	1	2	4	0.419
12	3	4	2	1	3	0.637
13	4	1	4	2	3	0.574
14	4	2	3	1	4	0.667
15	4	3	2	4	1	0.605
16	4	4	1	3	2	0.312
K_1_	2.129	1.913	1.462	2.66	2.568	​
K_2_	2.791	2.462	2.147	2.348	2.558	​
K_3_	2.554	2.771	2.847	2.216	2.333	​
K_4_	2.158	2.486	3.176	2.408	2.173	​
k_1_	0.532	0.478	0.366	0.665	0.642	​
k_2_	0.698	0.616	0.537	0.587	0.640	​
k_3_	0.639	0.693	0.712	0.554	0.583	​
k_4_	0.540	0.622	0.794	0.602	0.543	​
R	0.166	0.215	0.429	0.111	0.099	​

#### 2.2.4 E-nose

A PEN3.5 portable E-nose was used to detected the odor of LHEOs. Briefly, an aliquot of 40 μg of each LHEO sample was transferred into a headspace vial. The operating parameters of the E-nose were configured as follows: a sensor purge time of 300 s, an automatic zeroing time of 10 s, and a sample injection flow rate of 400 mL/min. The average sensor response values extracted from the stable period between 117 s and 119 s were utilized for subsequent statistical analysis. All measurements were performed in triplicate. The specific descriptive characteristics and target volatile compounds of the 10 sensors within the E-nose array are summarized in Table 3.

**TABLE 3:** Ten sensors used in E-nose and their substances for sensing.

Nos	Sensor	Sensibility
1	W1C	Aromatic components
2	W5S	Nitrogen oxides, broad range
3	W3C	Ammonia and aromatic components
4	W6S	Mainly hydrogen, selectively, (breath gases)
5	W5C	Alkanes and aromatic components
6	W1S	Propane, broad range
7	W1W	Sulfur organic compounds
8	W2S	Ethanol
9	W2W	Aromatic components and organic-sulfides
10	W3S	Propane (selective sometimes)

#### 2.2.5 Separation of volatile compounds in LHEO

The separation of volatile compounds was performed using a GC × GC-MS system. The chromatographic separation was achieved using a dual-column configuration: a primary column (^1^D) of HP-5MS Ultra Inert (36.57 m × 250 μm × 0.25 μm) and a secondary column (^2^D) of DB-17MS (2 m × 0.18 μm × 0.18 μm), coupled via a solid-state modulator. The injection port was maintained at 280 °C, and 1 μL of the sample was injected in splitless mode. The oven temperature program started at 50 °C (held for 5 min), then increased to 290 °C at a rate of 5 °C/min (held for 5 min). For the modulator, the modulation period was set at 8 s. The hot entry and exit zones followed the oven ramp, starting at 50 °C and 80 °C and reaching final temperatures of 290 °C and 320 °C, respectively. The cold zone initial temperature was 9 °C, decreased to −51 °C at 50 °C/min (held for 23.8 min), and then returned to 9 °C at 20 °C/min. Mass spectrometry (MS) was conducted using an Xtr-EI source with an electron energy of 70 eV. The temperatures of the transfer line, ion source, and quadrupole were set at 280 °C, 230 °C, and 150 °C, respectively. Data acquisition was performed in full scan mode across a mass range of 35–350 amu.

#### 2.2.6 Identification and quantitation of volatiles

The volatile compounds were tentatively identified by matching their mass spectra against the NIST17 library and further verified by comparing their linear retention indices (RI), calculated relative to a series of C7-30 n-alkanes, with literature values. Positive identification was achieved by comparing the retention times and mass spectra with those of authentic standards. Quantitative analysis was performed using the internal standard method, with 2,6-dichlorotoluene (0.65 mg/mL) serving as the internal standard to calculate the relative concentrations of the volatile constituents (Liang et al., 2023).

#### 2.2.7 Sensory evaluation

The aroma profile of LHEOs enhanced with various metal salts was determined in accordance with the modified version of GB/T 14454.2 2008. Eleven sensory panel composed of 6 males and 5 females, who were trained based on their ability to identify aromatic descriptors, was used to evaluate the six major herbal, floral, sweet, balsamic, woody and roasted notes. Evaluations were conducted in a standardized sensory laboratory with white light at a controlled temperature of 18 °C. Basically, the researcher labelled the LHEO samples using three-digit random codes. A 10-point linear scale was employed, ranging from 0 (not detectable) to 9 (extremely strong). The sensory scores from the 11 panelists were averaged to represent the final aroma intensity for each attribute.

#### 2.2.8 Statistical analysis

The software SPSS 27.0 (IBM, Armonk, NY, USA) was used for the determination of significant differences and the calculation of Pearson correlation coefficient values. SIMCA 14.1 (Umetrics, Umeå, Sweden) was utilized to perform orthogonal partial least squares discriminant analysis (OPLS-DA), along with the determination of variable importance in projection (VIP) values. TBtools were used to create the heatmaps and Origin 2021 (OriginLab, Northampton, MA, USA) line charts, bar graphs, radar charts and Venn diagrams.

## 3 Results and discussion

### 3.1 Results of single-factor experimental

**FIGURE 1:**
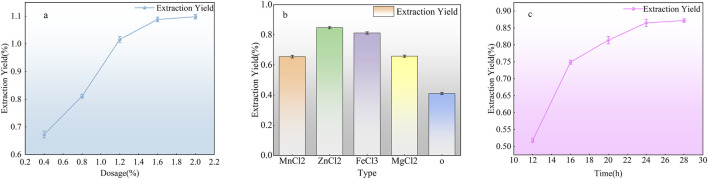
Effects of **(a)** salts dosage, **(b)** metal salts type, and **(c)** extraction time on the extraction yield of LHEO.

The dependence of essential oil yield on the FeCl_3_ concentration was illustrated in Figure 1a. A rapid, continuous increase in yield was observed as the FeCl_3_ concentration rose to 1.2%, followed by a distinct plateau where the growth rate significantly decelerated. This trend suggests a concentration-dependent effect on the structural degradation of the matrix; specifically, the capacity of hydronium ions within the FeCl_3_ solution to disrupt the lignocellulosic framework likely reaches a state of saturation beyond the 1.2% threshold. The analysis presented in Figure 1b demonstrated that all four types of metal salts increased the extraction efficiency. However, the control group (O) remains the lowest. The essential oil yield was roughly doubled when ZnCl_2_ and FeCl_3_ were added. It is suggested that metal ions can help hydrolyze biomass. As a result of catalytic action, complex biopolymers degrade to smaller molecular weight hydrocarbons, which are more likely to partition into the aqueous phase and steam entrainment (Wang et al., 2023). In terms of the kinetics of extraction (Figure 1c), the yield exhibited a gradual upward trend as the time of distillation was prolonged. Though extraction rates started to plateau after 24 h, indicating that LHEO recovery neared saturation point, after which time increments would result in diminishing returns.

### 3.2 Results of orthogonal experimental design

The optimization of extraction was carried out by using L_16_ (4^5^) orthogonal array based on single factor test. Metal salt type (A), salt dosage (B) and extraction time (C) were fixed as independent variables and yield of essential oil was taken as response index. Table 2 depicted the experimental results with range analysis. The range R value of each of the three factors is higher than that of the blank control group. This indicates that the three selected factors were effective and had a noticeable impact on essential oil yield and experiment results. The effect of extraction time, salt dosage and salt type on yield were measured using a central composite design and the relative effect of the three factors was determined in the order R_C_ > R_B_ > R_A_. The best combination of parameters was ZnCl_2_ as salt type, 1.2% salt dosage and extraction time of 24 h. Under these optimized parameters, the verification experiment, performed in triplicate, exhibited a 106% increase (more than doubling) in the essential oil yield.

ANOVA was carried out to assess the significance of each factor statistically (Table 4). According to the ANOVA, the sequence of effect of the factors is extraction time > salt dosage > salt type, which shows consistency with the results of range analysis. This suggested that while all four selected metal salts markedly enhanced the essential oil yield, the differential effects between the specific salt types were marginal.

**TABLE 4:** ANOVA table.

Factors	SS	Df	MS	F-value	P-value	Significant^a^
A	0.077	3	0.026	2.896	0.124	​
B	0.096	3	0.032	3.624	0.084	​
C	0.436	3	0.145	16.397	0.003	**
Error	0.053	6	0.009	​	​	​

^a^ “**” means *p* < 0.01.

### 3.3 Aroma profile of LHEO

**FIGURE 2:**
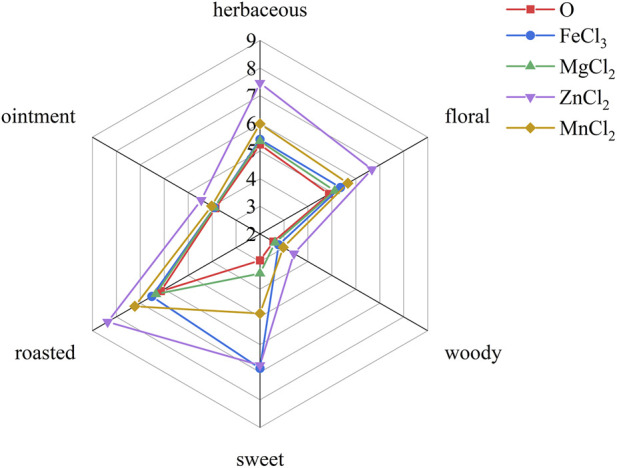
Aroma profile of LHEO treated by different metal salts.

Quantitative descriptive analysis (QDA) was employed to compare the differences in aromatic characteristics, including herbal, floral, woody, sweet, roasted, and balsamic notes, among LHEO extracted with various metal salt intensifiers, with the results illustrated in Figure 2. Essential oils intensifier with different metal salts showed different aroma characteristics as compared to the blank (O). Overall, although oil extracted with the Mg^2+^ aqueous solution improved all aromatic characters, the improvement was marginal. On the other hand, the essential oil with Fe^3+^ has a notably prominent sweet note while the herbal and floral notes remained unchanged. Both Mn^2+^ and Zn^2+^ treatments significantly enhanced all sensory attributes, with the intensification effect of Zn^2+^ being more pronounced than that of Mn^2+^. Based on the yield of essential oil and sensory assessment of LHEO, the best performer for intensified extraction was ZnCl_2_-based metal salt.

### 3.4 Results of E-nose analysis

**FIGURE 3:**
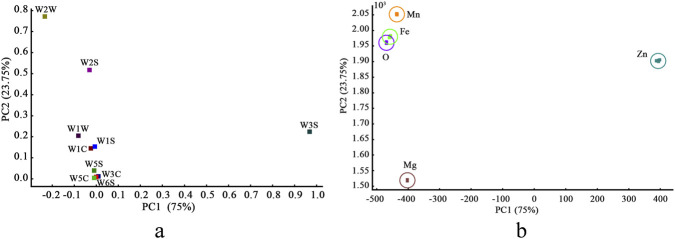
PCA **(a)** and LOA **(b)** plots of LHEOs treated with different metal salts.

The E-nose sensors exhibited high sensitivity to volatile compounds, with response intensities closely correlating with compound concentrations. Principal component analysis (PCA) was performed on the E-nose responses of LHEOs obtained via different metal salt-intensification strategies, as illustrated in the score plot (Figure 3a) and loading plot (Figure 3b). The first two principal components, PC1 and PC2, contributed 75% and 23.75% of the total variance, respectively, yielding a cumulative variance of 98.75%. This indicates that the PCA model captured the vast majority of the volatile profile information. Along the PC1 axis, LHEO samples from different metal salt treatments were clearly segregated. Specifically, the Zn^2+^-intensified sample was located on the positive axis of PC1, showcasing a distinct separation from the other groups. Along the PC2 axis, the control (untreated) sample and the Mg^2+^-intensified sample were positioned on the positive and negative sides, respectively, indicating a substantial difference between them, whereas the control, Fe^3+^, and Mn^2+^ groups clustered closely, suggesting highly similar volatile profiles. Loading analysis was further utilized to evaluate the discriminatory contribution of individual sensors to the principal components (Figure 3b). Sensor W3S exhibited the highest loading on PC1, while sensors W2W and W2S contributed most significantly to PC2. These findings demonstrate that the enhancement with different metal salts primarily induced significant variations in the concentrations of olefins and aromatic compounds within the LHEOs.

### 3.5 Volatile component analysis of essential oils extracted with different metal salts

A total of 235 compounds were identified across five samples (including the blank control), which were categorized into 11 classes: alcohols (32), aromatic hydrocarbons (5), phenols (31), aldehydes (28), acids (26), terpenes (32), ketones (29), alkanes (16), heterocycles (11), esters (24), and others (1). The number of compounds detected in each sample was 136 (O), 149 (Fe^3+^), 159 (Mg^2+^), 142 (Mn^2+^), and 149 (Zn^2+^) (Table 5). According to the results, the varieties and contents of volatile components in the essential oils of metal salts were higher than the blank control. The oil enriched with Mg^2+^ had the highest number of volatile compounds. With the application of Mn^2+^, the varieties remained largely unchanged but the concentrations were markedly increased. The presence of Fe^3+^ and Zn^2+^ contribute significantly to the variety, concentration and yield of volatile components. The essential oil extracted with Zn^2+^ had better sensorial performance, confirming Zn^2+^ as the best one among the metal salts tested. These results were in accordance with the results of the range analysis above.

**TABLE 5:** The composition of LHEOs extracted using various metal salt.

Sample	O		Fe3+		Mg2+		Mn2+		Zn2+	
	Sorts	Concentration/(µg/g)	Sorts	Concentration/(µg/g)	Sorts	Concentration/(µg/g)	Sorts	Concentration/(µg/g)	Sorts	Concentration/(µg/g)
Alcohols	17	5,910	23	5,530	23	46,200	19	6,748	20	7,222
Pahs	3	57.4	2	23.4	4	53.3	5	73.8	2	72.0
Phenols	18	1,484	21	731	23	1,500	20	977	16	1,376
Aldehydes	21	1,528	16	928	18	1,288	19	1828	21	1,519
Acids	15	8,124	22	15,012	21	12,263	14	7,939	19	10,800
Terpene	17	1,177	17	1,495	20	1,502	18	2,222	19	1775
Ketones	15	909	16	895	20	1,008	19	1,137	17	1,264
Alkanes	9	208	11	195	7	83.7	5	97.4	10	221
Heterocyclic ring	6	327	7	966	6	348.6	7	508	9	813
Esters	14	5,957	13	6,286	16	5,839	14	7,691	15	5,789
Others	1	40.7	1	28.3	1	23.3	2	134	1	52.7
Total	136	25,720	149	32,090	159	28,530	142	29,626	149	30,902

**FIGURE 4:**
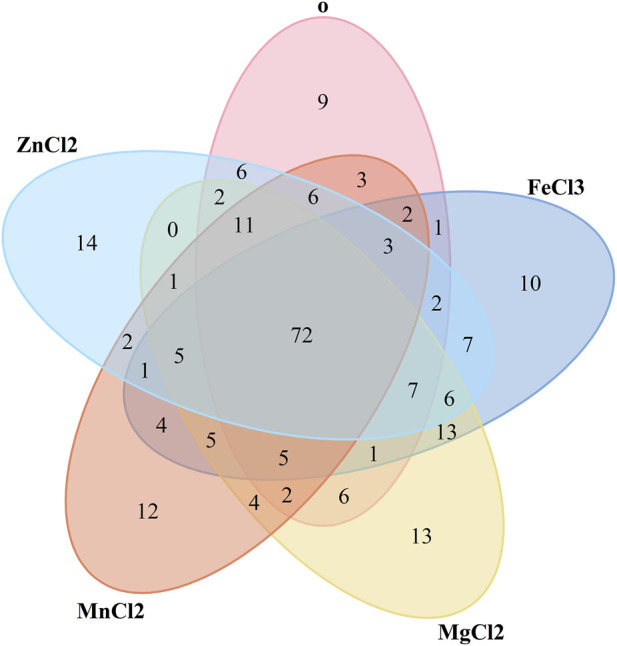
Venn diagrams of volatiles in LHEOs.

**FIGURE 5:**
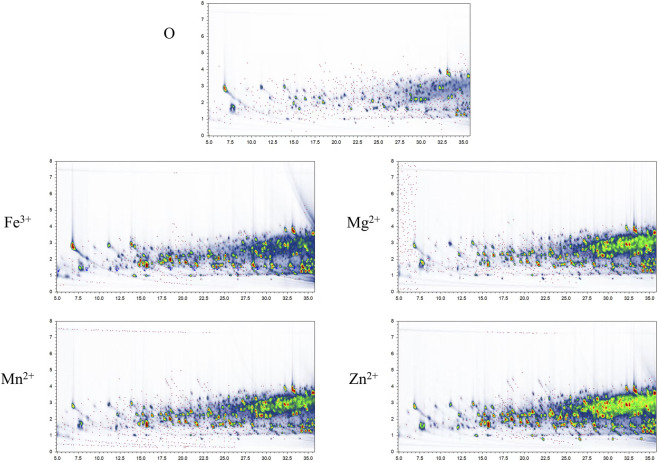
Two dimensional spectra of LHEOs treated with different metal salts.

As shown in Figure 4, a total of 72 volatile compounds were shared among the four intensifiers containing metal salts (Table 6), including 11 alcohols, one aromatic hydrocarbon, eight phenols, 11 aldehydes, nine acids, 10 terpenes, eight ketones, three alkanes, four heterocycles and seven esters. In addition, nine compounds like n-octanal, 3-methylbenzaldehyde, ocimene, cuparene, etc. were found in blank control (O) at a very low concentration. This indicated that the volatile components originally present on the site are not lost significantly due to the enhanced extraction process. Conversely, there were 97 compounds that were not present in the blank control (O) that were detected in the four metal salt-treated samples (Figure 5). The majority of these compounds have pleasant aroma properties of fruit and sweetness that can enhance LHEO aroma profile. The findings further support the feasibility of employing metal salt-intensified extraction for LHEO.

**TABLE 6:** Composition and relative content of volatiles in LHEO extracted with different metal salts.

Compound	NIST RI	RI^a^	Concentration (μg/g)					*p*	VIP^b^	Identification^c^
			O	FeCl_3_	MgCl_2_	ZnCl_2_	MnCl_2_			
Linalool	1,099	1,111	966 ± 8.75b	1,093 ± 4.84a	431 ± 14.2c	1,145 ± 47.3a	1,139 ± 30.4a	0.000	2.506	MS, RI, S
Benzeneethanol	1,116	1,125	36.5 ± 3.91d	50.3 ± 2.62b	49.3 ± 0.803b	56.5 ± 3.02a	42.7 ± 1.75c	0.000	0.478	MS, RI, S
Myrcenol	1,118	1,128	1.31 ± 0.942d	6.41 ± 0.721c	2.58 ± 0.942d	10.6 ± 0.541a	8.29 ± 0.153b	0.000	0.309	MS, RI, S
*α*-terpineol	1,190	1,197	367 ± 8.83c	413 ± 11.3b	163 ± 1.86d	530 ± 18.5a	520 ± 12.2a	0.000	1.686	MS, RI, S
Nerol	1,228	1,238	136 ± 8.77c	152 ± 0.521b	99.2 ± 3.12d	197 ± 7.41a	161 ± 6.80b	0.000	1.159	MS, RI, S
Geraniol	1,255	1,261	320 ± 12.9c	367 ± 0.539b	283 ± 7.96d	397 ± 23.3a	347 ± 11.6c	0.000	1.625	MS, RI, S
*β*-eudesmol	1,649	1,666	120 ± 4.76b	131 ± 12.4b	115 ± 7.39b	166 ± 11.6a	120 ± 3.82b	0.000	0.660	MS, RI, S
(*Z*)-9-Tetradecenol	1,666	1,671	21.0 ± 1.68b	4.61 ± 0.0290e	7.43 ± 0.158d	15.8 ± 1.62c	24.8 ± 0.512a	0.000	0.424	MS, RI, S
Isophytol	1948	1955	356 ± 8.40c	412 ± 10.7b	309 ± 9.05d	525 ± 25.1a	423 ± 24.6b	0.000	1.246	MS, RI, S
Phytol	2,114	2,128	3,226 ± 193a	1944 ± 20.8d	2,339 ± 149c	3,495 ± 215a	3,176 ± 9.43b	0.000	3.437	MS, RI, S
Geranylgeraniol	2,201	2,203	137 ± 1.40c	216 ± 5.84b	133 ± 10.9c	360 ± 23.7a	157 ± 13.0c	0.000	1.440	MS, RI, S
***Alcohols(11)***	​	​	**5,686**	**4,788**	**3,931**	**6,897**	**6,119**	​	​	​
3,4,5-Trimethoxytoluene	1,407	1,408	39.4 ± 0.893c	13.9 ± 1.16e	17.5 ± 0.424d	63.5 ± 1.41a	50.5 ± 1.44b	0.000	0.587	MS, RI, S
***PAHs(1)***	​	​	**39.4**	**13.9**	**17.5**	**63.5**	**50.5**	​	​	​
Phenol	981	1,002	39.0 ± 0.796d	50.8 ± 0.149b	44.3 ± 1.63c	53.1 ± 2.71a	55.4 ± 2.95a	0.000	0.469	MS, RI, S
2-Cresol	1,054	1,074	53.0 ± 3.72b	34.7 ± 2.50c	54.5 ± 0.325b	66.0 ± 3.45a	64.5 ± 2.80a	0.000	0.500	MS, RI, S
Paracresol	1,077	1,095	147 ± 7.02c	117 ± 3.61d	127 ± 8.52d	161 ± 1.26b	142 ± 3.21c	0.000	0.554	MS, RI, S
Guaiacol	1,090	1,099	44.1 ± 2.16b	31.9 ± 1.33c	40.8 ± 0.238b	56.2 ± 2.90a	40.5 ± 2.99b	0.000	0.417	MS, RI, S
2-Ethylphenol	1,140	1,163	122 ± 9.89b	18.2 ± 0.192c	115 ± 3.20b	271 ± 18.6a	22.6 ± 0.0520c	0.000	1.618	MS, RI, S
3-Methyl-4-ethylphenol	1,238	1,252	100 ± 6.60b	32.5 ± 1.70d	75.2 ± 2.65c	129 ± 3.95a	101 ± 7.29b	0.000	0.793	MS, RI
4-(1-Methylpropyl)phenol	1,315	1,338	51.3 ± 0.976c	14.4 ± 0.101d	80.2 ± 1.80b	83.2 ± 0.367b	125 ± 3.45a	0.000	1.070	MS, RI
2,4-Di-tert-butylphenol	1,514	1,521	14.6 ± 0.296d	23.0 ± 2.05a	15.1 ± 0.0740d	18.4 ± 0.360c	20.6 ± 0.360b	0.000	0.298	MS, RI, S
***Phenols(8)***	​	​	**571**	**323**	**552**	**838**	**572**	​	​	​
Diaethylacetal	728	759	434 ± 6.33b	406 ± 3.63b	333 ± 13.5c	324 ± 7.77c	616 ± 38.7a	0.000	1.877	MS, RI, S
(*E,E*)-2,4-heptadienal	1,012	1,022	5.40 ± 0.917b	6.29 ± 0.207a	6.44 ± 0.442a	5.00 ± 0.0150c	7.36 ± 0.703a	0.013	0.156	MS, RI, S
Decanal	1,206	1,211	36.3 ± 0.176c	8.78 ± 0.196e	21.7 ± 1.09d	42.1 ± 0.590a	40.2 ± 1.24b	0.000	0.495	MS, RI, S
Carvomenthenal	1,225	1,225	31.6 ± 1.70c	15.2 ± 0.164d	36.6 ± 0.911c	132 ± 6.04a	72.4 ± 6.90b	0.000	0.977	MS, RI
Hendecenal	1,297	1,303	135 ± 7.17a	21.6 ± 0.239d	84.9 ± 5.16c	117 ± 6.87b	126 ± 3.04a	0.000	0.932	MS, RI, S
Undecanal	1,307	1,313	263 ± 11.3b	70.5 ± 0.127d	287 ± 6.04a	209 ± 13.7c	248 ± 5.22b	0.000	1.334	MS, RI, S
Dodecanal	1,409	1,412	66.5 ± 2.97c	35.6 ± 0.146e	52.8 ± 1.01d	79.9 ± 3.40b	88.2 ± 4.93a	0.000	0.626	MS, RI, S
Tetradecanal	1,613	1,619	14.8 ± 1.13a	9.61 ± 0.239b	15.5 ± 1.78a	16.7 ± 0.661a	15.3 ± 2.06a	0.003	0.221	MS, RI, S
4-Biphenylaldehyde	1,662	1,667	114 ± 7.40c	66.8 ± 2.44d	113 ± 6.37c	185 ± 16.0a	136 ± 6.70b	0.000	0.903	MS, RI, S
1-Pentadecanal	1715	1719	57.0 ± 2.45c	48.4 ± 0.103d	65.1 ± 3.02b	80.5 ± 5.72a	87.5 ± 3.63a	0.000	0.645	MS, RI, S
Palmitaldehyde	1817	1822	104 ± 2.03b	83.8 ± 1.40c	53.8 ± 12.8d	154 ± 12.6a	99.4 ± 0.927b	0.000	0.806	MS, RI
***Aldehydes(11)***	​	​	**1,260**	**773**	**1,070**	**1,345**	**1,537**	​	​	​
Isovaleric acid	850	874	33.6 ± 2.04e	113 ± 7.44a	76.8 ± 3.79c	95.6 ± 4.87b	39.0 ± 0.633d	0.000	0.927	MS, RI, S
Methylethylacetic acid	861	882	16.7 ± 0.253e	72.4 ± 0.144b	53.6 ± 2.61c	78.3 ± 4.65a	42.2 ± 1.82d	0.000	0.845	MS, RI, S
Octanoic acid	1,180	1,188	7.69 ± 0.655d	14.6 ± 1.32c	21.8 ± 0.813a	18.9 ± 0.0460b	9.15 ± 0.294d	0.000	0.428	MS, RI, S
Hendecanoic acid	1,468	1,476	49.3 ± 5.30b	68.5 ± 0.737a	52.0 ± 3.00b	66.8 ± 3.56a	30.3 ± 1.82c	0.000	0.647	MS, RI, S
Tridecylic acid	1,664	1,671	93.7 ± 1.66c	140 ± 5.67b	90.7 ± 6.10c	196 ± 16.1a	43.3 ± 1.70d	0.000	1.236	MS, RI, S
Pentadecylic acid	1867	1835	40.9 ± 0.436c	58.9 ± 2.27b	24.9 ± 1.13d	80.7 ± 6.50a	34.7 ± 1.94c	0.000	0.693	MS, RI, S
Palmitic acid	1968	2032	2,177 ± 6.75a	2,142 ± 25.3a	2,151 ± 16.6a	2,128 ± 35.5a	2,167 ± 8.37a	0.233	0.628	MS, RI, S
Telfairic acid	2,133	2,165	3,017 ± 64.6a	3,041 ± 9.72a	3,058 ± 7.27a	3,017 ± 2.20a	3,045 ± 28.2a	0.643	0.543	MS, RI, S
Oleic acid	2,141	2,136	2,294 ± 36.4a	2,253 ± 3.43a	2,253 ± 35.1a	2,231 ± 5.45a	2,237 ± 29.2a	0.213	0.821	MS, RI, S
***Acids(9)***	​	​	**7,730**	**7,904**	**7,782**	**7,913**	**7,647**	​	​	​
*α*-cubebene	1,351	1,357	5.04 ± 0.510b	2.52 ± 0.150c	4.80 ± 0.851b	5.50 ± 0.392b	17.9 ± 0.547a	0.000	0.430	MS, RI
Copaene	1,376	1,382	69.3 ± 4.09d	34.2 ± 0.681e	90.7 ± 0.188c	106 ± 4.90b	246 ± 9.89a	0.000	1.576	MS, RI, S
1-Pentadecene	1,492	1,492	9.92 ± 0.836b	7.45 ± 0.303c	8.64 ± 0.593b	9.87 ± 0.889b	13.2 ± 0.984a	0.000	0.218	MS, RI, S
Calamenene	1,523	1,533	23.4 ± 0.681e	29.4 ± 0.0750c	26.7 ± 0.643d	37.2 ± 0.314b	39.5 ± 1.59a	0.000	0.447	MS, RI, S
*δ*-cadinene	1,524	1,532	23.7 ± 2.12d	34.0 ± 1.36c	40.1 ± 0.262b	17.1 ± 0.318e	48.5 ± 2.12a	0.000	0.641	MS, RI, S
Cariophyllene epoxide	1,581	1,596	57.7 ± 2.37d	115 ± 3.39a	46.8 ± 0.447e	80.1 ± 6.35c	104 ± 7.37b	0.000	0.853	MS, RI
*α*-hexadecene	1,592	1,596	17.7 ± 0.475c	22.4 ± 1.62b	23.8 ± 1.27b	42.4 ± 1.35a	10.3 ± 0.249d	0.000	0.567	MS, RI, S
Hexahydroaplotaxene	1,692	1,694	43.5 ± 2.10c	10.5 ± 0.989e	21.6 ± 0.552d	57.9 ± 4.37b	65.3 ± 0.196a	0.000	0.646	MS, RI, S
*α*-octadecene	1793	1795	21.9 ± 1.13c	16.3 ± 0.170d	12.9 ± 0.707e	28.9 ± 0.0170a	26.1 ± 0.901b	0.000	0.330	MS, RI, S
Neophytadiene	1838	1842	458 ± 0.675d	545 ± 2.52c	534 ± 33.0c	773 ± 34.7a	656 ± 14.9b	0.000	1.800	MS, RI, S
***Terpene(10)***	​	​	**730**	**817**	**810**	**1,157**	**1,226**	​	​	​
Indanone	1,292	1,290	8.83 ± 0.646b	7.68 ± 0.418c	8.44 ± 0.0900b	12.9 ± 0.752a	8.80 ± 0.227b	0.000	0.201	MS, RI, S
Damascenone	1,386	1,392	18.5 ± 0.983a	7.89 ± 0.128e	21.5 ± 1.22c	49.5 ± 0.554a	32.9 ± 0.365b	0.000	0.568	MS, RI, S
*β*-ionone	1,486	1,493	89.3 ± 0.552c	43.5 ± 1.32e	52.8 ± 0.945d	138 ± 2.96a	115 ± 7.21b	0.000	0.810	MS, RI, S
2-Tridecanone	1,496	1,498	11.6 ± 0.482b	7.59 ± 0.319c	11.2 ± 0.903b	16.0 ± 0.769a	16.2 ± 0.622a	0.000	0.270	MS, RI, S
2-Tetradecanone	1,597	1,602	16.5 ± 0.141c	16.0 ± 1.42c	10.7 ± 0.210d	23.5 ± 1.29a	18.7 ± 0.189b	0.000	0.280	MS, RI, S
2-Pentandecanone	1,698	1700	27.9 ± 1.15d	32.0 ± 0.575c	32.9 ± 0.524c	40.6 ± 0.630b	42.8 ± 0.366a	0.000	0.443	MS, RI, S
Fluorenone	1749	1753	119 ± 0.464b	68.5 ± 0.395d	91.7 ± 0.465c	209 ± 12.3a	121 ± 2.35b	0.000	0.992	MS, RI, S
Perhydrofarnesyl acetone	1844	1849	257 ± 7.34c	288 ± 5.05b	307 ± 20.7b	388 ± 12.8a	375 ± 8.43a	0.000	1.313	MS, RI, S
***Ketones(8)***	​	​	**548**	**471**	**536**	**878**	**731**	​	​	​
Farnesan	1,366	1,401	25.2 ± 0.220b	20.3 ± 0.494c	6.59 ± 0.776e	27.9 ± 1.42a	9.32 ± 0.946d	0.000	0.546	MS, RI, S
2,6,10-Trimethyl-tridecane	1,449	1,465	15.6 ± 0.542b	11.6 ± 1.12c	15.6 ± 0.912b	21.2 ± 1.08a	21.7 ± 1.95a	0.000	0.297	MS, RI
Phytan	1795	1802	27.4 ± 1.09c	30.3 ± 1.51c	20.6 ± 0.337d	45.4 ± 2.89a	40.2 ± 2.99b	0.000	0.433	MS, RI, S
***Alkanes(3)***	​	​	**68.3**	**62.2**	**42.8**	**94.5**	**71.3**	​	​	​
Fural	833	855	61.1 ± 1.44d	555 ± 20.4a	112 ± 9.68c	217 ± 6.32b	127 ± 5.13c	0.000	2.017	MS, RI, S
2-Acetylfurane	911	928	2.05 ± 0.233e	15.3 ± 0.128b	7.74 ± 0.644c	16.1 ± 0.150a	4.65 ± 0.584d	0.000	0.397	MS, RI, S
Linalool oxide	1,074	1,082	84.0 ± 5.99c	77.7 ± 2.17c	52.7 ± 4.14d	148 ± 8.06a	101 ± 3.83b	0.000	0.787	MS, RI, S
Dibenzofurane	1,515	1,528	171 ± 5.65c	54.8 ± 1.86e	124 ± 4.79d	264 ± 9.75a	192 ± 0.205b	0.000	1.158	MS, RI, S
***Heterocyclic ring(4)***	​	​	**318**	**703**	**296**	**645**	**424**	​	​	​
Methyl laurate	1,526	1,526	8.64 ± 0.739b	5.71 ± 0.457c	7.22 ± 0.989b	12.9 ± 0.752a	8.78 ± 0.823b	0.000	0.216	MS, RI, S
Ethyl laurate	1,594	1,602	5.42 ± 0.0660b	5.81 ± 0.387b	4.35 ± 0.781c	7.62 ± 0.867a	4.62 ± 0.145b	0.001	0.164	MS, RI, S
Methyl isocostate	1792	1796	137 ± 1.02c	147 ± 2.09c	291 ± 12.2a	194 ± 8.61b	182 ± 2.70b	0.000	1.360	MS, RI, S
Methyl hexadecanoate	1926	1929	428 ± 5.35a	425 ± 1.55a	430 ± 2.96a	429 ± 0.327a	424 ± 11.7a	0.813	0.174	MS, RI, S
Methyl linoleate	2093	2,106	859 ± 13.7a	853 ± 20.1a	855 ± 16.9a	850 ± 5.51a	854 ± 1.70a	0.979	0.089	MS, RI, S
Methyl linolenate	2099	2,114	1,058 ± 8.67b	1,087 ± 1.17a	1,087 ± 3.29a	1,080 ± 7.68a	1,083 ± 2.79a	0.002	0.646	MS, RI, S
Ethyl linolenate	2,169	2,180	2,160 ± 9.00a	2,146 ± 22.2a	2,152 ± 15.0a	2,175 ± 7.12a	2,156 ± 1.17a	0.314	0.343	MS, RI, S
***Esters(7)***	​	​	**4,519**	**4,522**	**4,535**	**4,555**	**4,531**	​	​	​
***Total(72)***	​	​	**21,471**	**20,377**	**19,573**	**24,386**	**22,908**	​	​	​

^a^ RI: Retention indices of compounds on DB-Wax column.

^b^ Obtained by OPLSDA, model.

^c^ Method of identification: MS, mass spectrum comparison using NIST20 library; RI, retention index in agreement with literature values; S: standard of volatiles.

Bold values mean the total concentrations of different classes of compounds.

The significantly high concentrations of alcohols, aldehydes, acids, terpenes, ketones, and esters shown by all the samples might work in synergy to develop the unique flavors of LHEO. Fatty acids such as oleic acid, linoleic acid, palmitic acid, and esters like methyl palmitate, methyl linoleate, methyl linolenate and ethyl linolenate have made up a greater than 50% of the total. However, these compounds possess very faint odors, typically characterized by subtle fatty notes or a near-absence of aroma (de Menezes et al., 2024), and their concentrations showed no significant changes following metal salt-intensified extraction.

Alcohols are major contributors to the overall fragrance and share odor structures with floral and fruity notes with intense aromas, widely abundance in flowers, fruits and herbs (Qu et al., 2024). After the enhancement by Zn^2+^, the total alcohol level in the LHEO increased by 21% (5,686 μg/g to 6,897 μg/g) with major increases in linalool, myrcenol, nerol and phytol. After intensification with different metal salts, concentrations of nearly all aldehydes and ketones increased. Furthermore, while heterocyclic compounds were less diverse and abundant, specific components such as furfural and 2-acetylfuran, representative compounds for sweetness, showed multi-fold increases in the intensified oils. This indicates that metal salts significantly facilitate the extraction of sweetening compounds from LHEO.

The remarkable variations in the chemical proportions and yields of essential oil components under different metal salt treatments can be systematically elucidated through three interconnected mechanisms. First, the strategic chemical disruption of the cell wall matrix via selective coordination plays a pivotal role. The botanical tissue possesses a dense, cross-linked macromolecular network (such as pectin and cellulose) that encapsulates the volatile oil glands. Metal cations, especially transition metal ions with vacant orbitals, can engage in selective coordination complexation with the oxygen-containing functional groups (hydroxyl and carboxyl groups) present in both the cell wall polysaccharides and specific volatile components. This interaction effectively weakens the biopolymer barriers, lowering mass transfer resistance and accelerating the liberation of matrix-bound compounds during SD (Zhang et al., 2026). Second, the variation is driven by the classical salting-out effect and competitive ionic hydration. Cations possess distinct ionic radii and charge densities, which directly govern their water-polarizing capabilities. Divalent and trivalent ions with high charge densities form tight hydration shells by competing for free water molecules. This severe reduction in available free water significantly decreases the solubility of hydrophobic volatile constituents (such as monoterpenes and sesquiterpenes) in the aqueous phase. As a thermodynamic consequence, the gas-liquid partition coefficients of these hydrophobic hydrocarbons are shifted toward the steam phase, altering the final oil profile (Hyde et al., 2017; Seto et al., 2007). Third, the localized alteration of phase equilibrium dynamics further modulates the component ratios. The accumulation of these dissolved metal salts inherently modulates the boiling characteristics and interfacial tension of the extraction mixture. This local thermodynamic shift fosters a more turbulent micro-boiling environment at the water-steam interface, facilitating the rapid co-evaporation of high-boiling-point characteristic aroma compounds (Iskăndărov et al., 1998).

To sum up, the different chemical constituents of LHEO contribute in synergism towards the overall aroma profile. The main volatile flavor compounds are alcohols, aldehydes, ketones, and terpenes. Furthermore, Heterocycles, acids, and esters support the main components. They enrich and modify the overall scent. Compared to the essential oil obtained via conventional steam distillation, those extracted through metal salt intensification exhibited varying degrees of increase in both the diversity and total concentration of volatile compounds.

### 3.6 Differential analysis of volatile compounds

**FIGURE 6:**
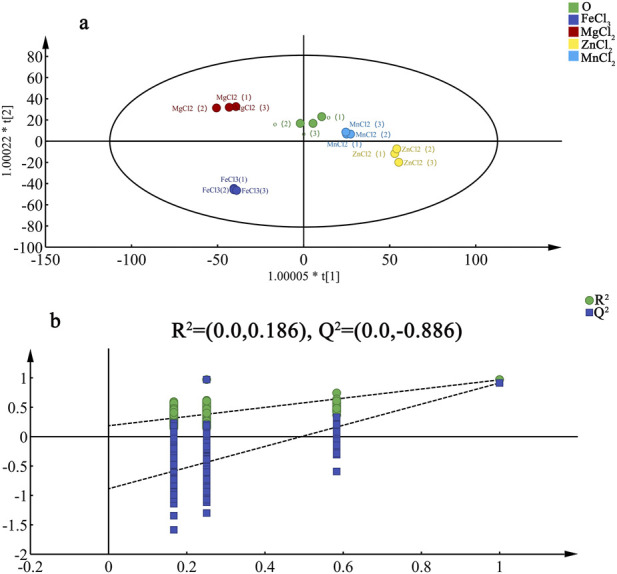
OPLS-DA score plots **(a)** and permutation test **(b)** of volatile compounds in LHEO with different metal salts processing methods.

To study the volatile profiles of LHEO enhanced by varied metal salts, the OPLS-DA model was built by taking metal salt type as independent variables and 72 common volatile compounds as dependent variable (Figure 6a). The score plot generated in the process indicates a deviation of the essential oils extracted with metal salts. The model had an R^2^X for independent variables value of 0.950, an R^2^Y for dependent variables value of 0.987, and a Q^2^ value of 0.969. Both R^2^ and Q^2^ values are significantly greater than 0.5. Thus, with high reliability and stability, and strong predictive capacity, the model is robust (Liang et al., 2025). A permutation test with 200 iterations (Figure 6b) was conducted to further validate the model. The results showed that the Q^2^ regression line intersected the Y-axis at the negative half-axis with an intercept of −0.886, confirming the validity of the model and the absence of overfitting (Yang et al., 2026). Thus, the model effectively discriminates between the essential oils intensified by different metal salts. Spatial analysis showed that MgCl_2_ was positioned in the second quadrant, FeCl_3_ in the third, and ZnCl_2_ in the fourth. Although both the blank control and MnCl_2_ were in the first quadrant, they were clearly separated into two distinct clusters. The clear separation of all five samples demonstrates significant differences in the volatile component concentrations among the essential oils extracted under different metal salt-intensified conditions.

### 3.7 Cluster analysis of differential volatile compounds

**FIGURE 7:**
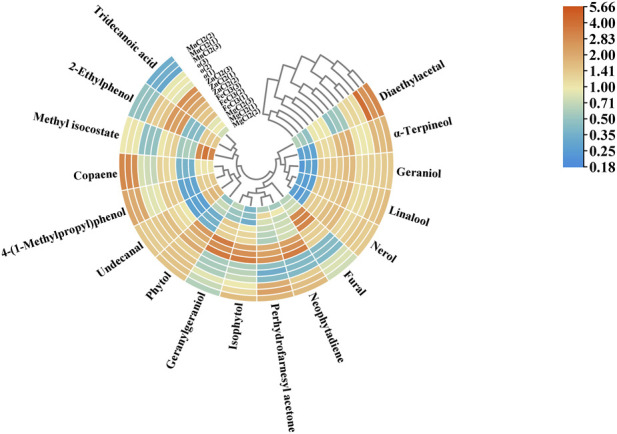
Heatmap visualization of differential compounds in LHEO treated with different metal salts.

To further characterize the variations in volatile profiles and identify key markers, 17 differential aromatic compounds, including seven alcohols, two phenols, two aldehydes, two terpenes, and one each of acid, ketone, ester, and heterocycle, were screened based on the criteria of P < 0.05 and VIP >1 (Yang et al., 2026). Cluster analysis was subsequently performed on these compounds, as illustrated in Figure 7. The results indicate that these 17 differential components effectively categorized the essential oils intensified by different metal salts into five distinct groups.

Compared to the blank control (O), the concentrations of furfural, phytone, geranylgeraniol, and neophytadiene were significantly increased in all metal salt-intensified samples. These compounds impart fresh sweet and fruity notes, suggesting that metal salts may facilitate the release of bound sweet-contributing volatile compounds in LHEO (Liu et al., 2026). Specifically, in the Mg^2+^ -intensified oil, only α-cubebene and methyl isoalantolactone showed slight increases, while most other compounds remained unchanged or even decreased, indicating that Mg^2+^ is less effective for intensification. In the Fe^3+^ treatment, the levels of α -terpineol, linalool, and geraniol were relatively stable compared to the blank, whereas furfural and geranylgeraniol contents rose markedly. For the Mn^2+^ treatment, a decrease was observed in components such as 2-ethylphenol and tridecanoic acid, while compounds providing sweet notes, such as furfural, neophytadiene, phytol, isophytol, and phytone, showed an upward trend.

Notably, chemical profiling revealed that only Zn^2+^ and Mn^2+^ elevated the overall levels of differential volatile metabolites across all treatment groups, with Zn^2+^ exhibiting a far more pronounced enhancement that particularly boosting nearly all components except acetal. This quantitative enrichment was consistently validated by qualitative flavor diagnostics, where both the professional sensory panel and objective electronic nose arrays confirmed that Zn^2+^-treated samples possessed the most robust aroma intensity and optimal flavor characteristics. Consequently, by integrating these flavor profiles with the baseline data from single-factor screenings and orthogonal matrices, the multi-dimensional evidence robustly demonstrates that Zn^2+^ exerts the superior metal-ion intensification effect, making ZnCl_2_ the most effective salt for LHEO extraction.

## 4 Conclusion

This study investigated the enhancement of the extraction of LHEO with different metal salts solutions. According to the results, all four metal salts can significantly increase the LHEO yield and volatile diversity. Of the salts tested, Zn^2+^ treatment yielded the best overall yield, compounds and concentration related to productivity. Although the compound diversity of Mg^2+^ was the highest, the overall enhancement in aromatics was negligible. Mn^2+^ enhanced both yield and sensory properties to a moderate degree, while Fe^3+^ specifically promoted acid and heterocyclic compounds (furfural). According to this work, ZnCl_2_ has proved to be the most effective intensifier for boosting the yield and sweet-floral quality of LHEO. Future research will explore non-volatile constituents to further elucidate the intensification mechanisms in botanical extractions.

## Data Availability

The raw data supporting the conclusions of this article will be made available by the authors, without undue reservation.
